# TNF*α* Affects Ciliary Beat Response to Increased Viscosity in Human Pediatric Airway Epithelium

**DOI:** 10.1155/2016/3628501

**Published:** 2016-11-29

**Authors:** Claudia González, Karla Droguett, Mariana Rios, Noam A. Cohen, Manuel Villalón

**Affiliations:** ^1^Department of Otorhinolaryngology, Faculty of Medicine, Pontificia Universidad Católica de Chile, Santiago, Chile; ^2^Department of Physiology, Faculty of Biological Sciences, Pontificia Universidad Católica de Chile, Santiago, Chile; ^3^Department of Otorhinolaryngology-Head and Neck Surgery, University of Pennsylvania, Philadelphia, PA, USA; ^4^Surgical Services, Philadelphia Veterans Affairs Medical Center, Philadelphia, PA, USA

## Abstract

In airway epithelium, mucociliary clearance (MCC) velocity depends on the ciliary beat frequency (CBF), and it is affected by mucus viscoelastic properties. Local inflammation induces secretion of cytokines (TNF*α*) that can alter mucus viscosity; however airway ciliated cells have an autoregulatory mechanism to prevent the collapse of CBF in response to increase in mucus viscosity, mechanism that is associated with an increment in intracellular Ca^+2^ level ([Ca^2+^]_i_). We studied the effect of TNF*α* on the autoregulatory mechanism that regulates CBF in response to increased viscosity using dextran solutions, in ciliated cells cultured from human pediatric epithelial adenoid tissue. Cultures were treated with TNF*α*, before and after the viscous load was changed. TNF*α* treatment produced a significantly larger decrease in CBF in cultures exposed to dextran. Furthermore, an increment in [Ca^2+^]_i_ was observed, which was significantly larger after TNF*α* treatment. In conclusion, although TNF*α* has deleterious effects on ciliated cells in response to maintaining CBF after increasing viscous loading, it has a positive effect, since increasing [Ca^2+^]_i_ may prevent the MCC collapse. These findings suggest that augmented levels of TNF*α* associated with an inflammatory response of the nasopharyngeal epithelium may have dual effects that contribute to maintaining the effectiveness of MCC in the upper airways.

## 1. Introduction

Mucociliary clearance (MCC) is a critical defense mechanism as it removes microbes and contaminants from the airway. The epithelium of the upper and lower airways system is composed of ciliated cells, whose ciliary beating removes the mucus layer that covered the epithelium. The MCC velocity is determined by ciliary beat frequency (CBF) and viscosity of the mucus layer [1]. Bacterial and viral infections negatively impact MCC through multiple processes such as induction of local inflammation, recruitment of neutrophils [2], secretion of cytokines [3], and alteration of mucus viscosity [4]. In patients with cystic fibrosis (CF), the airway surface becomes severely dehydrated, with an increase in mucus viscosity, which is not easily transported by cilia [5].

Tumor necrosis factor alpha (TNF*α*) is a proinflammatory cytokine produced primarily by cells of hematopoietic origin, including myeloid lineage such as monocytes and macrophages [6]. This cytokine exerts pathophysiological roles, affecting calcium homeostasis in the different tissues including neurons [7], cardiomyocytes [8], and smooth muscle cells [9, 10]. In the airways, TNF*α* can be released by bronchial epithelial cells in inflammatory conditions [11]; therefore several studies have focused on this cytokine in attempts to establish its role in the pathogenesis of respiratory diseases. TNF*α* concentration is significantly higher in patients with asthma [12], chronic rhinosinusitis [13, 14], and CF [15] compared to normal patients. In CF airways, TNF*α* stimulates fluid secretions by submucosal glands by a mechanism that involves CF transmembrane conductance regulator [15]. A continuous local production of TNF*α* within the olfactory mucosa in chronic rhinosinusitis patients results in a progressive inflammation with olfactory loss [16]. An enhanced expression of several inflammatory mediators such as TNF*α* has been demonstrated in alveolar macrophages of human with chronic heart failure, to be involved in mechanism such as pulmonary vascular congestion [17]. In severe refractory asthma, TNF*α* is able to prolong eosinophils survival by inhibiting apoptosis and thus exacerbating the pathology [18]. Several studies have shown that TNF*α* affect CBF in the airways. In bovine bronchial ciliated cell cultures [19], human nasal ciliated epithelial cells [20], human sinus epithelial cells cultures [21], and murine trachea epithelial cells [22] CBF showed an increment or a decrease in CBF depending on the concentration of TNF*α* used and the experimental model. However, no studies have shown evidence of TNF*α* effect upon calcium homeostasis in epithelial cells from the airways.

The effectiveness of MCC is affected by inflammatory conditions where mucin overproduction and hypersecretion are induced [23]. It has been demonstrated that TNF*α* induces mucin secretion from guinea pig trachea epithelial cells after an 8 h of treatment (10 to 15 ng/mL) [24]. These treatment conditions were similar to others studies, where TNF*α* stimulate mucin secretion by human airways epithelium [25] and by rat tracheal epithelial cell cultures [26]. However, ciliated cells have a functional autoregulatory mechanism that prevents the collapse of mucus transport that maintains the CBF, in response to changes in viscosity to which they are normally exposed [27]. This mechanism, described in the ciliated epithelium of hamster oviduct [28], frog esophagus [29], and rabbit trachea [27], has been shown to be locally generated within the cell. This autoregulatory mechanism relies on cells to maintain CBF under high viscosity conditions, allowing ciliated epithelia to adjust their CBF to changes in viscous load, without collapsing MCC. This mechanism is coupled to an increase in [Ca^2+^]_i_ through the activation of the transient receptor potential vanilloid 4 channel, which produces an increment of [Ca^2+^]_i_ by the release of this ion from intracellular stores at lower viscous load (2–37 cP, 2–15% dextran) or the entry of calcium from extracellular space at high viscous load (37–200 cP, 15–30% dextran) [28].

The simultaneous effect of changes in mucus viscosity and high levels of proinflammatory factors, like TNF*α*, had not been investigated on ciliary activity. The hypothesis is that TNF*α* alters intracellular calcium homeostasis affecting the autoregulatory response of ciliated cells. In the present study, we used primary cultures of human pediatric epithelial adenoid tissue to evaluate the effect of TNF*α* on the response mechanism to viscous overload and calcium homeostasis in the control of CBF.

## 2. Material and Methods

### 2.1. Tissue Samples

Adenoid tissues were obtained from pediatric patients (3–12 years) undergoing adenoidectomy for obstructive pathology (adenoid or adenotonsillar hypertrophy) with parental informed consent. The study design and informed consent were reviewed and approved by the Ethics Committee of Pontificia Universidad Católica de Chile.

Immediately after acquisition, adenoid tissue was placed in Hank's balanced salt solution (HBSS, Sigma-Aldrich, St Louis, MO, US, H1387) pH 7.4, supplemented with antibiotics (10 *μ*g/mL streptomycin, 100 U/mL penicillin G, and 0.125 *μ*g/mL amphotericin B; Life Technologies/Gibco BRL, NY, US).

From each adenoid sample, we obtained around 6 cultures, each one with 4 or 5 explants surrounded by a monolayer of ciliated cells. For the purpose of this study, we used 48 cultures of ciliated cells, obtained from 22 pediatric patients. At least three different patients were used for each experimental group.

### 2.2. Primary Cultures of Adenoid Tissue

Primary cultures, to yield a monolayer of epithelial cells explants, were performed as described previously [30]. Briefly, adenoid tissue was placed in a DMEM/F12 medium with pronase (P5147, Sigma-Aldrich) 0.05% w/v and left overnight at 4°C. Next day, the epithelium was mechanically removed, cut into 2–4 mm pieces, and soaked in NHS medium [30]. The pieces of epithelium were placed onto coverslip and covered with a sterile dialysis membrane in Rose chamber which were filled with 2 mL of NHS medium containing 10% heat inactivated horse serum (Biological Industries, Israel). Cultures were maintained in an incubator at 37°C and were ready to be used when a monolayer of ciliated cells is observed.

### 2.3. Western Blot Analysis of TNFR1 and TNFR2 Expression

Cultured cells were collected and frozen in PBS 1x at −70°C until use. Total proteins were extracted by homogenization in RIPA buffer (150 mM NaCl, 50 mM Tris-HCl, pH 7.5, 1% Triton X, 0.5% Na deoxycholate, and 1 mM PMSF) as described previously [31]. Protein concentration of homogenates was measured and 30 *μ*g was separated by 12% SDS-PAGE and transferred to nitrocellulose membrane (MSI, Westboro, MA, US). Membranes were preincubated in blocking buffer (5% nonfat milk 0.05% Tween 20 in Tris-buffered saline 20 mM (Tris-HCl 4 mM and NaCl 100 mM; pH 8)) for 1 h at room temperature and incubated overnight at 4°C with polyclonal rabbit anti-TNF-R1 (H-5, Santa Cruz Biotechnology Inc, CA, US) or TNF-R2 (D-2, Santa Cruz) antibody diluted 1 : 5000 in Tris-buffered saline/0.1% Tween-20 (TBST). Following this, membranes were incubated with anti-rabbit secondary antibody coupled with horseradish peroxidase for 1 h at room temperature (diluted 1 : 10,000 in TBST). Bound antibody was detected by chemiluminescence using the Western Lightning ECL system (NEN, Western Lightning, PerkinElmer, CA, US) and quantified by densitometry. A common tissue sample was included on each gel to allow for standardization of chemiluminescence levels and exposure times. Staining of each gel (posttransfer) and membrane with Coomassie Brilliant Blue (Sigma Chemical Co., St Louis, MO, US) assessed the accuracy of sample loading and the efficiency of protein transfer. HeLa cells were used as a positive control.

### 2.4. CBF Measurements

CBF was monitored and recorded by performing microphotodensitometry according to a procedure described previously [32]. Briefly, the spectral structure of light-scattering fluctuations produced by the moving cilia of a single cell was detected with a photodiode, and the signal was processed online using a digital spectrum computer card (model r360; Rapid System, Jersey City, NJ, US). CBF data in individual cells are expressed as a percentage of basal CBF (mean ± SEM) to normalize the results.

### 2.5. Intracellular Calcium Levels Measurements

Intracellular calcium levels ([Ca^2+^]_i_) were determined using a spectrofluorometric technique described previously [30]. Cultures of ciliated cells were loaded with 1.5 *μ*M Fura-2AM (Invitrogen Corp NY, US) for 1 h at 37°C. The fluorescence of individual cells was detected at room temperature with an Olympus fluorescence microscope coupled to an image acquisition system (Metafluor, Universal Imaging Corporation, v6.1). Images were acquired at excitation wavelength of 340 and 380 nm and detected at 510 nm.

### 2.6. Experimental Procedure

All cultures used in the present study were observed with a Nikon Diaphot inverted microscope with a 40x objective lens. After 7 days of culture, cells showed spontaneous ciliary activity with a frequency range of 8–12 Hz. Cultures were treated with human recombinant TNF*α* (10 ng/mL) (Sigma Chemical Co.) or control solution during 24 or 48 hours. After this time, ciliated cells were equilibrated in HBSS at 35°C for a period of 5 min, while ciliary activity was continuously monitored to determine average basal activity. Viscosity was modified by the addition of dextran (500,000 MW, United States Biological, MA, US) to the culture medium. Solutions were prepared at 10% (14.4 cP) or 20% (73 cP) dextran. After 25 min of CBF recordings under increased viscosity, cultures were washed three times with HBSS medium to completely remove dextran.

To determine the source [Ca^2+^]_i_ increment associated to the effect of TNF*α* on CBF response to increase viscous loading, cultures were previously incubated for 48 hours with TNF*α* (10 ng/mL). Then cultures were treated with either Thapsigargin 2.5 *μ*M (Calbiochem-Novabiochem International, San Diego, CA, US) for 30 min or Gadolinium 100 *μ*M (G7532, Sigma Chemical Co) for 5 min, previous to the change in viscosity of the medium. Thapsigargin was dissolved in dimethyl sulfoxide (DMSO) at a final concentration of 0.1%. At this concentration, DMSO had no effect on CBF.

### 2.7. Data Analysis

Statistical comparisons between different experimental conditions were made by analysis of variance of the area under the curve of arcsine-transformed data using Prism 6 (GraphPad Software, San Diego, CA, US), followed by a Bonferroni's multiple comparison test. The criterion for a significant difference was a final value of *p* < 0.05. Data are expressed as means ± SE; *n* refers to the number of cultures analyzed.

## 3. Results

### 3.1. Basal CBF Was Not Affected by TNF*α* Treatment

**Figure 1:**
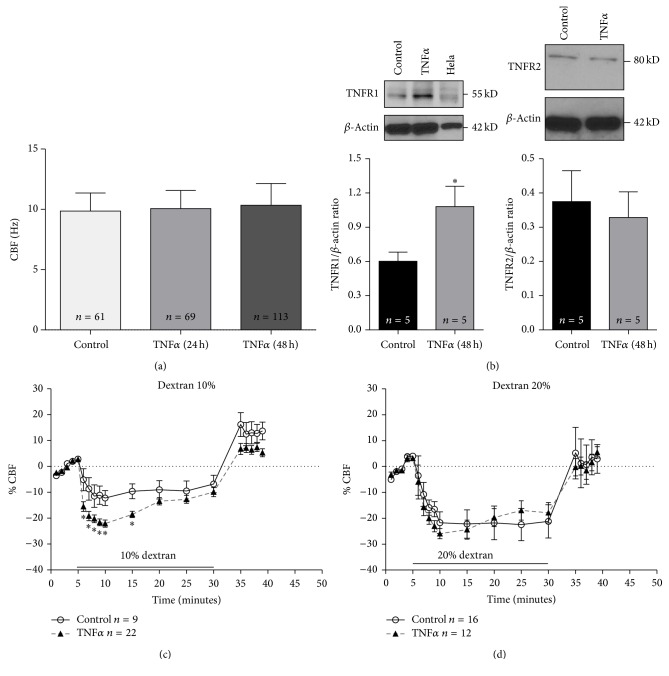
TNF*α* incubation reduces the ciliary beat frequency (CBF) response after viscous loading. (a) Basal CBF in cultures treated with TNF*α* for 24 or 48 hours. No statistically differences between treatments were found. (b) Ciliated cell cultures were treated for 48 hours with TNF*α* (10 ng/mL) and subjected to western blot analyses for TNF-R1, TNF-R2, and *β*-actin. The quantification of the immunoreactive band expressed as ratio of *β*-actin band showed that the treatment with TNF*α* significantly increases the expression of TNF-R1 in airway ciliated cells. *n* = 5; ^*∗*^ *p* < 0.05.* Inset*. The immunoreactive band of TNF-R1 increased in cultures treated with TNF*α* compared with control. HeLa cells were used as positive control. ((c) and (d)) Time course response of CBF changes, expressed as a percentage (%) of baseline CBF, in primary cultures of ciliated cells after 48 hours of treatment with TNF*α* (10 ng/mL) or control solution (Hank's, 1 cP) before and after low viscous (10% dextran) (c) or high viscous (20% dextran) (d) loading. Graph represents the mean ± SEM for different treatments. ^*∗*^Statistically significant differences between control and TNF*α* using test-*t* comparing each point of CBF (*p* < 0.05). *n* corresponds to individual ciliated cell measure from three patient.

To evaluate the effect of TNF*α* on CBF, primary cultures of pediatric adenoid tissue were treated with TNF*α* for 24 or 48 hours. Basal CBF mean was 9.85 ± 1.5 Hz in control cultures (*n* = 61), 10.07 ± 1.5 Hz in 24 hours in treated cultures (*n* = 69), and 10.34 ± 1.8 Hz in 48 hours in treated cultures (*n* = 113). No statistically differences between treatments were found (Figure 1(a)).

### 3.2. The Expression of TNF*α* Receptors in Cultured Ciliated Cells (TNF-R1 and TNF-R2) Was Changed by TNF*α* Treatment

Two TNF*α* receptors are activated by TNF*α*, being TNF-R1 the most common since it is expressed in the majority of cells and TNF-R2 is only limited to the immune cells [22]. However both receptors can be regulated independently and are biologically active [33]. Primary cultures from adenoid tissue were treated with TNF*α* for 48 hours. After this time, the expression of TNF*α* receptors was determined by western blot analysis. A single immunoreactive band of approximately 55 kDa was revealed, consistent with molecular weight previously reported for TNF-R1 (Figure 1(b), inset). TNF-R1 protein expression increased significantly following treatment with TNF*α* (Figure 1(b)). A band of 80 kDa for TNF-R2 was detected; however no changes in TNF-R2 protein expression were observed after TNF*α* treatment (Figure 1(b)).

### 3.3. CBF Response to Viscous Load Was Modified by TNF*α* Treatment

To determine the effect of TNF*α* on CBF response after increasing the viscous load of ciliated cells, cultures were treated with TNF*α* for 48 hours before culture viscosity was changed from control medium to 10% or 20% dextran solution. In control cultures, a decrease in CBF was observed immediately after the viscosity of the medium was increased, with a greater decrease in CBF in cultures exposed to 20% dextran. When cultures, previous to viscosity change, were treated with TNF*α*, CBF decreased furthermore only in culture exposed to 10% dextran. This effect of TNF*α* occurs in the first 10 min of viscous load then; TNF*α* treated cells seem to adjust the CBF to control values. After 25 min with dextran solution, cultures were washed to remove dextran and CBF had a slight increment over basal values (Figures 1(c) and 1(d)). The average CBF-decrease in cultures (compared to baseline) exposed to 10% dextran was 17.03 ± 1.13% in TNF*α* treated cultures (*n* = 22) and 9.28 ± 3.4% in control (*n* = 9) (Figure 1(c)). However, when cultures were exposed to 20% dextran, CBF decreased equally in control cultures (16.84 ± 4.4%, *n* = 16) and in cultures treated with TNF*α* (18.98 ± 2.34%, *n* = 12) (Figure 1(d)).

### 3.4. [Ca^2+^]_i_ Response to Viscous Overload Was Increased by TNF*α* Treatment

**Figure 2:**
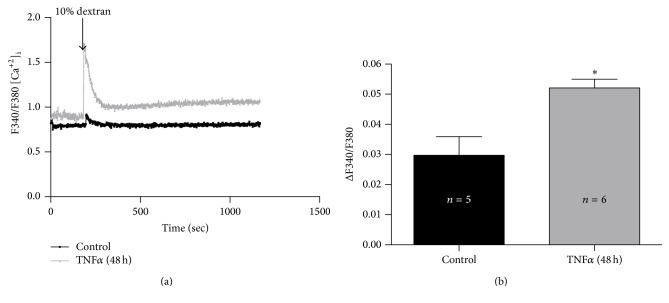
TNF*α* incubation increases [Ca^2+^]_i_ response after viscous loading. (a) Time course of the [Ca^2+^]_i_ after increasing the viscosity of the media with 10% dextran in primary cultures pretreated for 48 hours with TNF*α* (10 ng/mL) or control. (b) Difference between maximal [Ca^2+^]_i_ and basal calcium levels after 10% dextran solution or control. Each bar represents the mean ± SEM. ^*∗*^Statistically significant differences (*p* < 0.05). *n* corresponds to the number of culture used.

[Ca^2+^]_i_ was measured in cultures exposed to 10% dextran and pretreated for 48 hours with TNF*α*. A slight increase in [Ca^2+^]_i_ was observed after viscosity of the cultures was changed, which was significantly higher in cultures pretreated with TNF*α* (Figures 2(a) and 2(b)).

### 3.5. Calcium Homeostasis Mediates the CBF Response to Changes in Viscosity Exposed to TNF*α*

**Figure 3:**
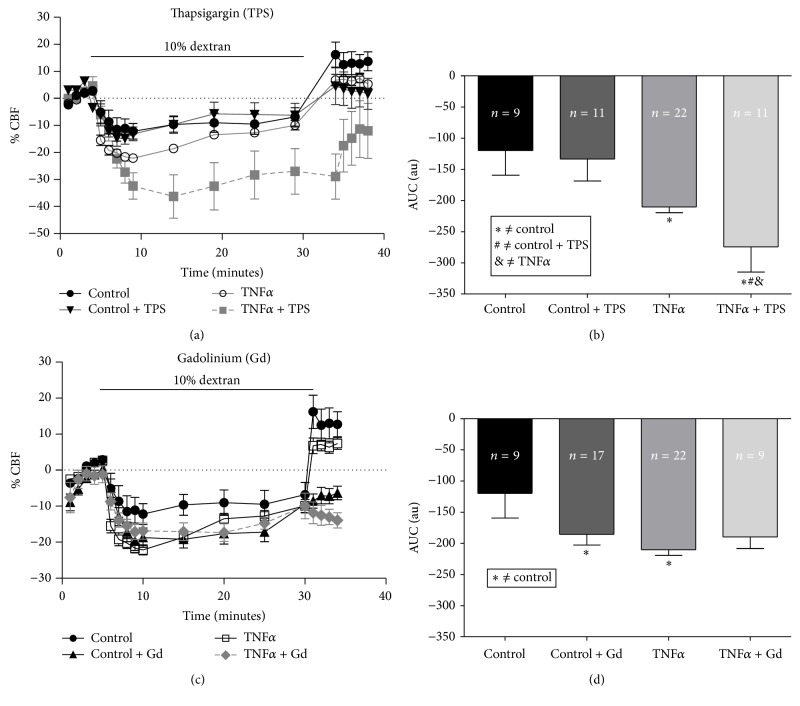
TNF*α* incubation affects intracellular calcium levels to prevent the collapse of CBF in response to viscous loading. ((a) and (c)) Time course of the changes in CBF in ciliated cells after 48 hours of treatment with TNF*α* (10 ng/mL) or control solution (HBSS) exposed to 10% dextran in the presence or absence of intracellular (Thapsigargin 2.5 *μ*M) (a) or extracellular calcium (Gadolinium 100 *μ*M) (c) blockers. ((b) and (d)) Area under the curve of (a) (b) and (c) (d) between 5 and 30 min of treatment. Each bar represents the mean ± SEM for different treatments. ^*∗*#&^Statistically significant differences (*p* < 0.05). *n* corresponds to individual ciliated cell measure from three patient.

To evaluate the effect of viscous loading and TNF*α* on the source of [Ca^2+^]_i_ increment, we cotreated cultures exposed to 10% dextran with TNF*α* and Thapsigargin, an inhibitor of the endoplasmic reticulum Ca-ATPase calcium pump, or Gadolinium, a Ca^2+^ channel blocker. In control cultures CBF was not affected by Thapsigargin treatment, showing an average CBF-decrease of 9.8 ± 3.3% equivalent to control CBF value. While cultures were cotreated with TNF*α* and Thapsigargin, CBF showed a statistically significant decrease (25.2 ± 6.2%) compared to cultures treated only with TNF*α* (17.03 ± 1.13%) or to controls (9.28 ± 3.4%), when the viscosity of the medium was increased (10% dextran) (Figures 3(a) and 3(b)).

When control cultures, exposed to 10% dextran, were treated with Gadolinium, a larger decrease in CBF (15.92 ± 2.29%) was observed compared to control cultures (9.28 ± 3.4%). Similar decrease of CBF was observed in cultures pretreated with TNF*α* with or without Gadolinium (14.55 ± 2.44% and 17.03 ± 1.13%, resp.), when the viscosity of the medium was changed (Figures 3(c) and 3(d)).

## 4. Discussion

In the present study, we demonstrated that human nasopharyngeal pediatric airway ciliated cells culture exposed to TNF*α*, a proinflammatory cytokine, produced a significant decrease in CBF response to changes in viscous loading (10% dextran), but has a positive effect, since we observed an increase in [Ca^2+^]_i_ that may prevent the MCC collapse. Although, the changes in CBF observed after increasing the viscosity in the presence of TNF*α* represent a decay of up to 30% of the CBF with respect to basal CBF, it is well established that MCC velocity is directly dependent on CBF and that small changes in CBF have a substantial effects on MCC effectiveness [34].

Long exposure (24 or 48 h) to a concentration of TNF*α* (10 ng/mL) did not affect basal CBF in human adenoid culture of ciliated cells. The concentration of TNF*α* used in our study is equivalent to the amount measured in bronchoalveolar fluid of human patients with acute respiratory distress syndrome, where TNF*α* concentration can reach ranges above 10 ng/mL [35]. In previous studies using human nasal explants mucosa, the exposure to TNF*α* (10 ng/mL) increased CBF after 24 h of incubation, returning to basal CBF after 48 h of exposure [20]. On the contrary, in human sinus epithelial cells cultures, the same concentration of TNF*α* (10 ng/mL) decreased CBF after 24 hours [21]. Although, the direct effect of TNF*α* upon CBF is still controversial, depending on the cellular context, the evidence suggests that this cytokine may have a potential role in a defense mechanism by directly affecting CBF in different models [20] or activating biochemical pathways such as nitric oxide which have been shown to increase MCC in the airways [21, 36].

Two receptors have been described for TNF*α*: the constitutive is p55 (TNF-R1) and the less abundant is p75 (TNF-R2) [31]. Ciliated cells expressed both subtypes, showing an upregulation only of TNF-R1 after 48 hours of TNF*α* incubation. The increase of TNF receptors might be necessary to sustain an acute and chronic defense reaction of the epithelium, in response to an airway injury. Similar results were obtained in mouse trachea, where TNF*α* increased only TNF-R1 mRNA and this receptor was exclusively located in tracheal epithelium [22]. Furthermore, in adult human heart the presence of both receptors has been shown, but the negative inotropic effects of TNF*α* appear to be initiated only by activation of TNF-R1 [37].

Human adenoid primary cultures were exposed to increase viscous loads that caused in both cases (10 and 20% dextran solution) a CBF reduction that was viscosity dependent. However, when cultures prior to the viscous load were incubated with TNF*α*, CBF decreased furthermore, only in cultures exposed to 10% dextran. No further decrease of CBF was observed with TNF*α* in cultures exposed to 20% dextran solution. Previous studies have demonstrated that CBF gradually dropped within the range of 2–15% dextran solution but at higher viscosities, in the range of 15–30% dextran solutions, CBF remained stable [28]. Our results corroborate that human ciliated cells have an autoregulatory mechanism which maintain CBF, preventing the collapse of MCC, allowing mucus transport in ciliated epithelial surfaces at high viscous conditions. Furthermore, TNF*α* did affect the autoregulatory response at the low viscosity range, mechanism that depends on the release of calcium from intracellular reservoir. However, it did not affect the autoregulatory response at the high viscosity range that requires an oscillatory influx of calcium from the extracellular space [28]. These observations suggest that although TNF*α* has an adverse effect on CBF at low viscosity range, the existence of an autoregulatory mechanism of ciliated cells in the airways prevents CBF from decreasing further than ~30% of the basal ciliary activity even at a wider viscosity range.

In cultures loaded with 10% dextran solution, the presence of TNF*α* induced an additional decrease in CBF compared to control group that lasted only 10 min. At higher viscosities, CBF decreased and remained reduced as long as the viscous load was present. This decrease-recovery response observed after increased loading with 10% dextran suggests the existence of a compensatory mechanism to restore CBF. Similar results reported by Johnson et al. [27] showed that at low viscosities CBF transiently decreased and then slowly recovered ciliary activity.

In this study, cultures exposed to an increased viscosity (10% dextran) produced a slight increment in [Ca^2+^]_i_ that it was significantly augmented after incubation with TNF*α* for 48 hours. In airway smooth muscle cells, it has been shown that TNF*α* increased [Ca^2+^]_i_ by modifying mitochondrial calcium concentration [38], decreasing the expression of Ca^2+^-ATPase, and slowing Ca^2+^ reuptake [39]. Furthermore, it has been demonstrated that TNF*α* induced the upregulation of protein kinase pathways leading to an increment in Ca^2+^ sensitivity [40]. Our results suggest that the increase in [Ca^2+^]_i_ observed after TNF*α* incubation could protect cells from a worse cilia slowing and the recovery of CBF to basal ciliary activity, after viscous loading.

To further investigate the effect of viscous loading and TNF*α* on the source of the rise in [Ca^2+^]_i_, we monitored changes in CBF in cultures exposed to 10% of dextran and TNF*α* in the presence of an inhibitor of calcium uptake (Thapsigargin) or a calcium channel blocker (Gadolinium). Our results showed that Ca^2+^ from the cytosolic reservoir and the extracellular influx contribute to maintaining the augmented levels of [Ca^2+^]_i_, to prevent a further decrease of CBF induced by the additive effect of viscous loading and TNF*α*. The combined action of TNF*α* and 10% dextran on CBF on ciliated cells from adenoid tissue seem to simulate the effect of high range viscosity (20% dextran), where the autoregulatory mechanism depends mainly on extracellular calcium influx [28].

After 25 min of viscous loading, culture medium was replaced to normal viscosity resulting in an increment of CBF compared to the previous basal level, probably due to the mechanical stimulation induced by replacing the medium. In cultures treated with Gadolinium, CBF remained diminished despite the mechanical stimulation. This observation confirmed the participation of extracellular calcium entry in the increase in CBF induced by mechanical stimulation, a mechanism that has been demonstrated in mouse epithelial cells [41]. The mucus secretion stimulated by TNF*α* is dependent on phospholipase C and protein kinase C (PKC) pathways, involving activation of nitric oxide synthase (NOS), generation of nitric oxide (NO), production of cGMP, and activation of cGMP-dependent protein kinase (PKG) [42], pathway that also stimulates ciliary beating through an interplay between nitric oxide pathway and elevated [Ca^2+^]_i_ [36, 43, 44].

In conclusion, our findings suggest that, during acute inflammation, where mucus viscosity and secretion of cytokines in airway are increased, TNF*α* has a negative effect upon the response of human nasopharyngeal pediatric airway ciliated cells to viscous loading. However, TNF*α* incubation also has a beneficial effect, since it induces an increment in [Ca^2+^]_i_ that contributes to maintaining the effectiveness of MCC in the upper respiratory tract. Further studies are required to advance our understanding of the mechanism of intracellular calcium homeostasis affected by TNF*α*, in order to prevent the collapse of MCC observed in chronic airway diseases.

## Abbreviations

CBF: Ciliary beat frequency
MCC: Mucociliary clearance.
